# Using eHealth to Support COVID-19 Education, Self-Assessment, and Symptom Monitoring in the Netherlands: Observational Study

**DOI:** 10.2196/19822

**Published:** 2020-06-23

**Authors:** Thomas Timmers, Loes Janssen, Joep Stohr, J L Murk, M A H Berrevoets

**Affiliations:** 1 Interactive Studios Rosmalen Netherlands; 2 Radboud University Medical Center Radboud Institute for Health Sciences IQ Healthcare Nijmegen Netherlands; 3 Máxima Medical Center Veldhoven Netherlands; 4 Elisabeth Twee Steden Hospital Tilburg Netherlands

**Keywords:** patient education, COVID-19, smartphone, mobile phone, self-management, eHealth, mHealth

## Abstract

**Background:**

The coronavirus disease (COVID-19) situation demands a lot from citizens, health care providers, and governmental institutions. Citizens need to cope with guidelines on social interaction, work, home isolation, and symptom recognition. Additionally, health care providers and policy makers have to cope with unprecedented and unpredictable pressure on the health care system they need to manage. By providing citizens with an app, they always have access to the latest information and can assess their own health. This data could be used to support policy makers and health care providers to get valuable insights in the regional distribution of infection load and health care consumption.

**Objective:**

The aim of this observational study is to assess people’s use of an app to support them with COVID-19 education, self-assessment, and monitoring of their own health for a 7-day period. In addition, we aim to assess the usability of this data for health care providers and policy makers by applying it to an interactive map and combining it with hospital data. The secondary outcomes of the study were user’s satisfaction with the information provided in the app, perceived usefulness of the app, health care providers they contacted, and the follow-up actions from this contact.

**Methods:**

This observational cohort study was carried out at the nonacademic teaching hospital “Elisabeth Twee Steden” (ETZ) in Tilburg, Netherlands. From April 1, 2020, onwards ETZ offered the COVID-19 education, self-assessment, and symptom tracking diary to their already existing app for patient education and monitoring.

**Results:**

Between April 1 and April 20, 2020, a total of 6194 people downloaded the app. The self-assessment functionality was used abundantly to check one’s health status. In total, 5104 people responded to the question about severe symptoms, from which 242 indicated to suffer from severe symptoms. A total of 4929 people responded to the question about mild symptoms, from which 3248 indicated to suffer from these. The data was successfully applied to an interactive map, displaying user demographics and health status. Furthermore, the data was linked to clinical data. App users were satisfied with the information in the app and appreciated the symptom diary functionality. In total, 102 users reached out to a health care provider, leading to 91 contacts.

**Conclusions:**

Our study demonstrated the successful implementation and use of an app with COVID-19 education, self-assessment, and a 7-day symptom diary. Data collected with the app were successfully applied to an interactive map. In addition, we were able to link the data to COVID-19 screening results from the hospital’s microbiology laboratory. This data could be used to support policy makers and health care providers to get valuable insights in the regional distribution of infection load and health care consumption.

**Trial Registration:**

Netherlands Trial Register NL8501; https://www.trialregister.nl/trial/8501

## Introduction

### Background

In December 2019, an outbreak of the coronavirus disease (COVID-19) began in Wuhan, China. As of April 25, 2020, the virus had been identified in 185 countries, accounting for 2.4 million confirmed cases and over 17,000 confirmed deaths [1]. On February 26, 2020, the first documented case in the Netherlands was registered at the Elisabeth Twee Steden (ETZ) Hospital in the city of Tilburg [2], and at the time of writing, there were 46,952 confirmed cases and 5,990 confirmed deaths in the Netherlands [3].

The COVID-19 situation demands a lot from citizens, health care providers, and governmental institutions. Citizens, for instance, need to cope with guidelines on social interaction, work, home isolation, and symptom recognition [4]. In a new situation, such as this pandemic, being knowledgeable about what severe symptoms are, how to measure them, and how to act according to the latest guidelines could be considered a challenge for many of them. Additionally, health care providers and policy makers have to cope with unprecedented and unpredictable pressure on the health care system they need to manage.

Electronic health (eHealth) offers a potential powerful means to support all stakeholders in situations like these. By providing citizens with an app, they always have access to the latest trustworthy information and education, and can be actively notified of important changes by a push notification [5]. Furthermore, the app allows them to track their symptoms and gather valuable data for both the users themselves, as well as health care providers and policy makers, who can use this data on a more aggregated level to assess the local or regional health status and (expected) pressure on the health care system [6-10]. The effective use of eHealth has been demonstrated before in the management of chronic diseases and other treatments [11-15].

### Objectives

The aim of this observational cohort study is to assess people’s use of an app to support them with COVID-19 education, self-assessment, and monitoring of their own health for a 7-day period by using a symptom diary. In addition, we aimed to assess how this data would be useful for health care providers and policymakers by applying it to an interactive map and combining it with hospital data.

## Methods

### Study Design

This observational cohort study was carried out at the nonacademic teaching hospital ETZ in Tilburg, Netherlands. On April 1, 2020, ETZ added the COVID-19 education, self-assessment, and symptom tracking diary to their already existing app for patient education and monitoring, called ETZ Treatment Guide (in Dutch: “ETZ Behandelwijzer”). This app is based on the Patient Journey App (Interactive Studios) and is normally used to support patients during their treatment by providing them with timely information. The app contains educational information for indications such as knee replacement surgery, breast cancer, and cataract. In close collaboration with other Dutch hospitals, the COVID-19 pathway was developed and added to the app. This led to a unique combination of functionalities (education, self-assessment, and symptom diary) and content (national guidelines and local implementation in the hospital). At the time of writing, the app is being used by over 15 hospitals in the Netherlands, Belgium, and Germany, accumulating over 30,000 downloads. This research focuses on the data collected in the ETZ area.

The app is fully stand-alone; in other words, it is not connected to health care providers or electronic health records. The app is free of charge and publicly available through the Apple App Store and Google Play store. ETZ patients that were already using the app, received an in-app message about the COVID-19 pathway. Other patients and inhabitants of the Tilburg area were informed through ETZ’s own website and several online and offline press releases. This study focuses on the downloads, users, and their actions in the first 2 weeks after the initial press release about the availability of the COVID-19 pathway in the ETZ app. We followed the Strengthening the Reporting of Observational Studies in Epidemiology (STROBE) guidelines and the STROBE Checklist for observational research [16].

### Informed Consent and Ethical Considerations

Once users choose the COVID-19 pathway in the app, they were informed about the fact that the hospital could use their data on an anonymous basis for research purposes. None of the questions asked in the app were mandatory. There were no indicators of substantial risk as a function of participating in this study. The study was registered at the Netherlands Trial Registry, with the reference number 8501. The study was reviewed by the Medical Ethics Committee of the Máxima Medical Center (Veldhoven, Netherlands) with the reference number N20.039, who judged that the Medical Research Involving Human Subjects Act (in Dutch: WMO) was not applicable.

### Participant Selection

All ETZ patients and residents of the Tilburg area were able to download the app from app stores. Participants needed to have a smartphone or tablet and were required to be fluent in Dutch to understand the content, as the app was only available in Dutch.

### Information and Functionality of the App

The app started with welcoming users to the COVID-19 guide by means of text and a video. In addition, users were told about the (default) option to receive push notifications and were encouraged to share the app with family and friends as well. Consecutively, the app showed a video on how to prevent the virus from spreading, provided by the Dutch Health Authority (RIVM; Rijksinstituut voor Volksgezondheid, Welzijn en Sport) and a link to RIVM’s website for the latest information. Changes in the RIVM’s mitigation strategies were updated in the app, to assure users always had the same guidelines available. Before users continued, they were informed about the fact that the information in the app might not apply to them or that the information in the app cannot replace the personal advice they receive from a health care professional.

Next, users were requested to answer three questions that would provide the hospital with information about the users themselves (age and gender) and their geographical location (4 digits of their postal code) to assess whether geolocation data could be used during outbreaks such as with COVID-19.

**Figure 1:**
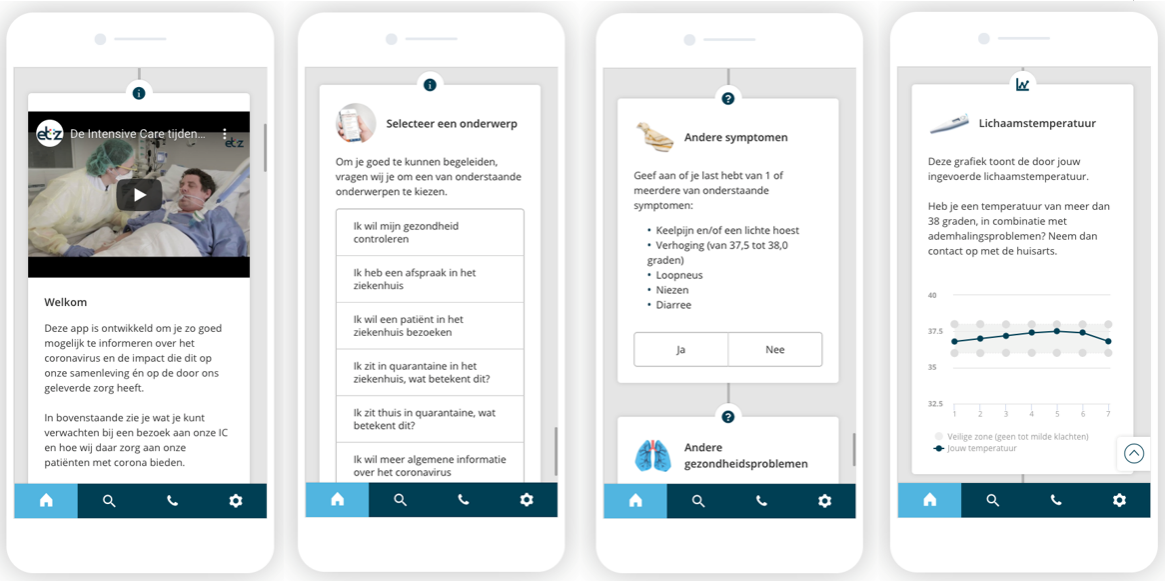
Examples of the coronavirus disease guide in the Elisabeth Twee Steden Behandelwijzer app (in Dutch). From left to right: the welcoming of patients to the app (including a video of the intensive care unit), the main menu to choose the type of information or functionality, part of the self-assessment tool (mild symptoms and underlying diseases are displayed), and the result of the 7-day tracking of symptoms (progression of body temperature is displayed).

After these introductory steps, the app provided a choice between six topics: I want to check my health, I have an appointment in the hospital, I want to visit or accompany a patient, I am in isolation at the hospital, I am in isolation at home, and I want general information about COVID-19 (Figure 1).

The “*I want to check my health*” part of the app offered users the possibility to self-assess their current COVID-19 health status, focusing on severe symptoms (body temperature of 38° Celsius or more, in combination with shortness of breath), mild symptoms (sore throat, coughing, rise in temperature from 37.5° to 38° Celsius, runny nose, sneezing, and diarrhea), and underlying diseases such as chronic obstructive pulmonary disease, asthma, diabetes, or heart failure. Based on the input the user provided, an outcome and advice on how to handle it was presented. This decision tree was based on the guidelines from the health authorities.

After the completion of the self-assessment, users were provided the possibility to keep track of their symptoms (body temperature and shortness of breath) for a 7-day period. When they chose to do so, they were requested to fill in the date of the first day of symptom tracking and were educated on when and how to measure and report the symptoms. They were informed once again that there was no connection between the app and the hospital to make sure users did not expect a response from ETZ health care staff. Users received daily push notifications at 10 AM to report their body temperature and at 2 PM to report their shortness of breath.

The other five parts of the app focused on educational content. Patients who wanted information about their appointment in the hospital got hospital specific information about planned consultations and surgeries. Patients who wanted to visit or accompany a patient in the hospital were provided the local guidelines on, for instance, visiting hours and the maximum number of visitors or people that could accompany a patient. Information about isolation in the hospital came from the hospital itself whereas the information on self-isolation and general COVID-19 information came from the RIVM’s website. All information in the app was presented in Dutch.

### Study Outcomes

The primary outcome of the study was the number of users that completed the COVID-19 self-assessment and the number of users that used the symptom diary. In addition, we aimed to assess the usability of this data for health care providers and policy makers by applying it to an interactive map and combining it with hospital data. The data users provided was divided into three regions, based on postal code regions: Tilburg City Area, Greater Tilburg Region, and other. The secondary outcomes of the study were user’s satisfaction with the information provided in the app, perceived usefulness of the app, health care providers they contacted, and the follow-up actions from this contact. All outcomes were measured by self-reported in-app questionnaires (Textbox 1).

Study outcomes were measured at baseline (user characteristics), after users indicated to want to self-assess their health on a daily basis for 7 days after using the health monitor (body temperature and shortness of breath), and on the seventh day after the start of the symptom diary to assess satisfaction, added value, contact with health care providers, and follow-up actions (Table 1).

**Overview of used questions per outcome.**

**User characteristics**

- What is your gender (Female, Male, other)?
- What is your age (numerical value between 18 and 110)?
- What are the first 4 digits of your postal code (numerical value)?

**Self-assessment/quick scan (based on the national Dutch Health Authority [RIVM] guidelines for coronavirus disease [COVID-19] symptom screening)**

- Do you suffer from a combination of fever (body temperature of 38.0° Celsius or more) and shortness of breath (yes/no)?
- Do you suffer from any of these mild symptoms: sore throat, mild cough, raise in body temperature (between 37.5 and 38.0° Celsius), runny nose, sneezing, or diarrhea (yes/no)?
- Do you have any underlying diseases such as chronic obstructive pulmonary disease, asthma, diabetes, or heart failure (yes/no)?

**Body temperature (based on the national RIVM guidelines for COVID-19 symptom screening)**

- Numeric value, one decimal, ranging from 35.0 to 45.0

**Shortness of breath (based on the national RIVM guidelines for COVID-19 symptom screening)**

- Numeric Rating Scale (NRS), 0-10 scale; 0 stands for “no trouble breathing at all,” 5 stands for “some troubles,” and 7 or higher stands for “trouble breathing even when in bed or on a couch.”

**Satisfaction with information in the app (self-developed questions, specifically for the study)**

- How satisfied are you with the information in this app? NRS, 0-10 scale; 0 stands for “not satisfied at all” and 10 stands for “extremely satisfied.”

**Added value of symptom monitoring using the app (self-developed questions, specifically for the study)**

- Do you find it valuable to monitor your health through this app? NRS, 0-10 scale; 0 stands for “not valuable at all” and 10 stands for “super valuable.”

**Contact with health care providers (self-developed questions, specifically for the study)**

- Did you contact a health care provider in the last 7 days? Multiple choice question: no contact with health care providers, contact with general practitioner, contact with hospital, or contact with general practitioner and hospital.

**Follow-up actions after contact with health care provider (self-developed questions, specifically for the study)**

- Was there a follow-up action as a result of your contact with the health care provider? Multiple choice question: none, visit to general practitioner, visit to hospital/emergency department, admittance to hospital, or admittance to intensive care unit.

**Table 1:** Overview of outcomes per measurement.

Measurement	Baseline	Day 1	Day 2	Day 3	Day 4	Day 5	Day 6	Day 7
User characteristics	✓	N/A^a^	N/A	N/A	N/A	N/A	N/A	N/A
Self-assessment/quick scan	✓	N/A	N/A	N/A	N/A	N/A	N/A	N/A
Body temperature	N/A	✓	✓	✓	✓	✓	✓	✓
Shortness of breath	N/A	✓	✓	✓	✓	✓	✓	✓
Satisfaction with information	N/A	N/A	N/A	N/A	N/A	N/A	N/A	✓
Added value of symptom monitoring	N/A	N/A	N/A	N/A	N/A	N/A	N/A	✓
Contact with health care providers	N/A	N/A	N/A	N/A	N/A	N/A	N/A	✓
Follow-up actions after contact with health care provider	N/A	N/A	N/A	N/A	N/A	N/A	N/A	✓

^a^Not applicable.

### Study Size

We did not perform an a priory sample size calculation but rather looked at the number of downloads the app had in other hospital regions where it was used. In those regions, the number of downloads was between 1000 and 2000 downloads per region. Given the population size in the Tilburg area and the press release ETZ was planning, we aimed at approximately 2000 downloads.

### Statistical Methods

Categorical variables are presented as numbers and percentages. Continuous variables are presented as means (with SD). There was no statistical comparison between groups, since the aim of this study is to assess the use of the app and the usability of the data that comes from it, rather than demonstrating in-between group differences. Descriptive statistics will be presented at postal code level. Data from the health self-assessment will be presented in tabular format. Data from the symptom tracking diary (body temperature and shortness of breath) will be represented in tabular format as well as line graphs. Data plotting for geolocation purposes was performed using raw data and the Google Maps and Google Geolocation application programming interface (API; Google). All data was analyzed using IBM SPSS Statistics for Macintosh, version 25.0 (IBM Corp).

## Results

### Study Sample

**Figure 2:**
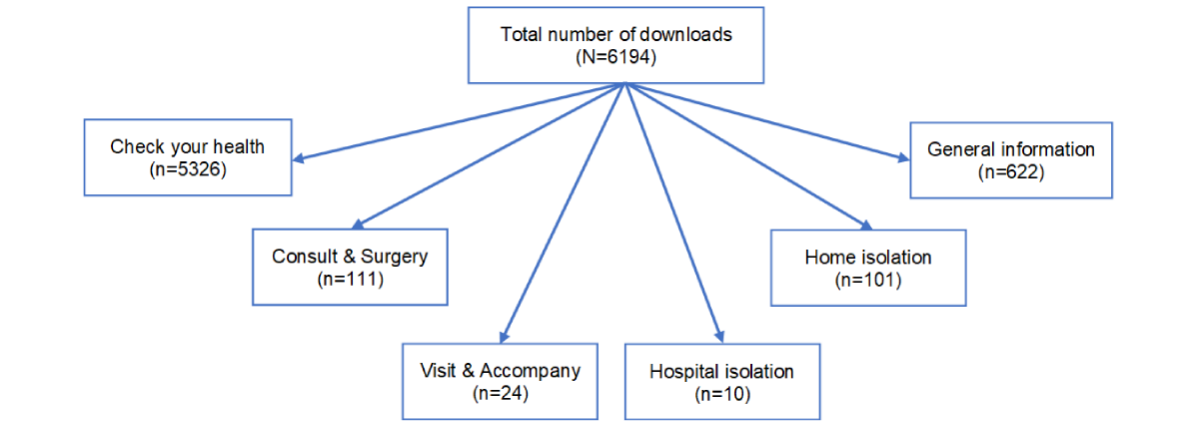
Overview of the functionalities of the app and the number of users that used them.

Between April 1 and April 20, 2020, a total of 6194 people used the app (5698 new downloads [92%], 496 existing app users [8%]). The average number of push notifications sent per day during this period increased from 113 to 561 (496%) and the average number of views per day increased from 178 to 611 (343%). The functionality that was used most often was the health self-assessment, which was used by 5326 users (86%). Figure 2 provides an overview of the functionalities of the app and the number of users that used them.

### User Characteristics

Of the 6194 downloads, 5364 (87%) users of the app shared data about their gender, and 5328 (86%) shared data about their age. The average age was 50.87 years (SD 14.38), and 46% (n=2455) of users were male. Within the group of users that checked their health (n=4655), the average age was 50.60 years, and 47% (n=2198) of users were male (Table 2).

Related to postal codes, a total of 5872 users (85%) reported the first 4 digits of their postal code. Postal code segments were used to divide the app users into 3 groups: Tilburg City (n=1464), Tilburg Region (n=1459) and Other (n=2949).

**Table 2:** User characteristics.

Group and characteristic			User
**Overall (n=6194)**			
	**Sex, n**		5364
		Male, n (%)	2455 (46)
		Female, n (%)	2884 (54)
	Age (n=5328), mean (SD)		50.87 (14.38)
**Users that checked their health (n=5326)**			
	**Sex, n**		4655
		Male, n (%)	2198 (47)
		Female, n (%)	2436 (53)
	Age (n=4624), mean (SD)		50.60 (14.42)

### Primary Outcomes

#### Use of the Self-Assessment Functionality

The self-assessment functionality was used abundantly to check user’s health status. In total, 5154 people responded to the question about severe symptoms, from which 242 (4.7%) indicated that they suffered from severe symptoms. A total of 4929 people responded to the question about mild symptoms, from which 3248 (65.9%) indicated that they suffered from these. Finally, 2929 people responded to the question concerning underlying diseases, and 1099 (22.3%) of them indicated to suffer from them. There were only small differences between the results reported by inhabitants of Tilburg City, the Tilburg Region, and the other users (Table 3).

**Table 3:** Self-assessment results.

Region	Severe symptoms	Mild symptoms (yes/no)	Underlying diseases (yes/no)
Tilburg City, n/N (%)	53/1178 (4.5)	751/1146 (65.5)	293/1144 (25.6)
Tilburg Region, n/N (%)	40/1111 (3.6)	664/1055 (62.9)	229/1055 (21.7)
Other, n/N (%)	149/2865 (5.2)	1833/2727 (67.2)	577/2721 (21.2)
Total, n/N (%)	242/5154 (4.7)	3248/4928 (65.9)	1099/4920 (22.3)

#### Use of the 7-Day Symptom Monitoring Diary

**Figure 3:**
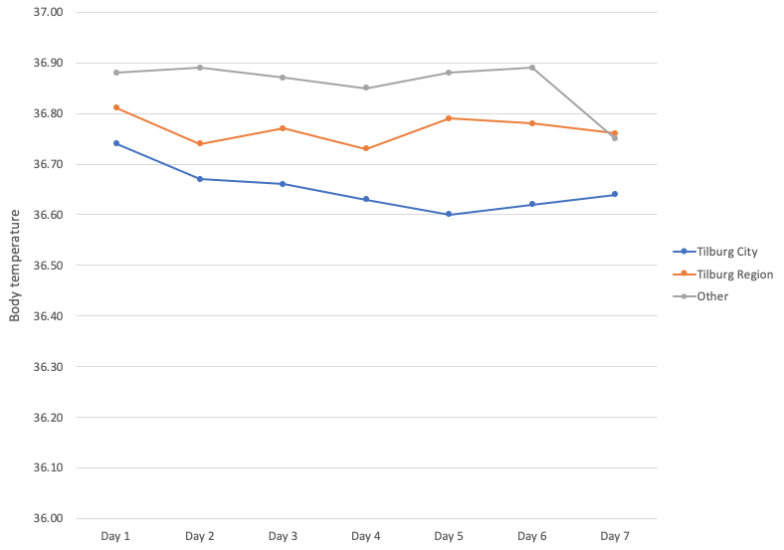
Body temperature results per day over a 7-day period as reported by inhabitants of Tilburg City, the Tilburg Region, and other areas.

The 7-day symptom diary was initially used by 1378 people. The data provided by users in the three groups is consistent in terms of the outcome (body temperature or shortness of breath) and standard deviation. There was a decrease in the number of users that completed the body temperature diary for 7 consecutive days, but this decrease was more pronounced in users from the “Other” area (642 on day one compared to 137 [21%] on day seven) than it was in Tilburg City (378 on day one compared to 119 [31%] on day seven) or Tilburg Region (338 on day one compared to 116 [34%] on day seven; Figure 3; see Multimedia Appendix 1 for all body temperature data). The same pattern is demonstrated in the shortness of breath diary, where the decrease was also more pronounced in users from the “Other” area (405 on day one compared to 83 [21%] on day seven) than it was in Tilburg City (239 on day one compared to 85 [35%] on day seven) or Tilburg Region (212 on day one compared to 72 [34%] on day seven).

**Figure 4:**
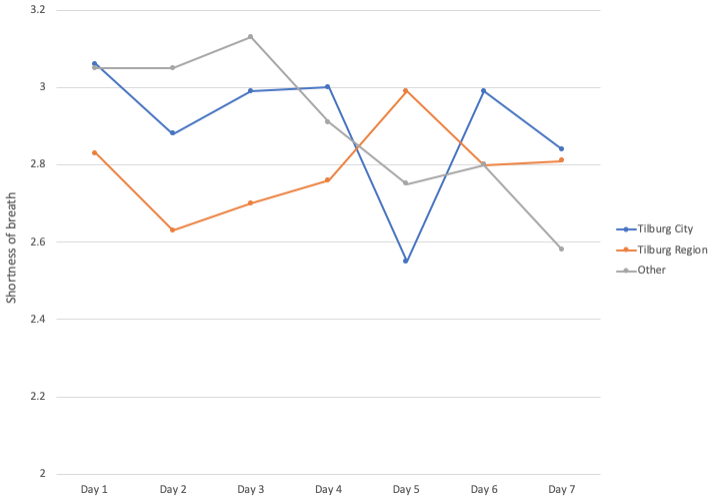
Shortness of breath results per day over a 7-day period as reported by inhabitants of Tilburg City, the Tilburg Region, and other areas.

In the “Other” group, there were 405 users that completed the seventh day of the diary on day one compared to 83 (20%) on day seven of all cases, compared to 239 users on day one to 85 [32%] on day seven in Tilburg City, as well as in the Tilburg Region (Figure 4; see Multimedia Appendix 1 for all shortness of breath data).

#### Usability of the Data for Health Care Providers and Policy Makers

The data that was gathered during the use of the app focuses on 4 domains: demographic information (age and gender), geographical information (postal code), COVID-19 symptoms, and contact with health care providers. Combining this data creates an overview of symptoms per postal code, to which clinical data from the ETZ about patients that have been tested positive for COVID-19 can be added (Table 4).

**Figure 5:**
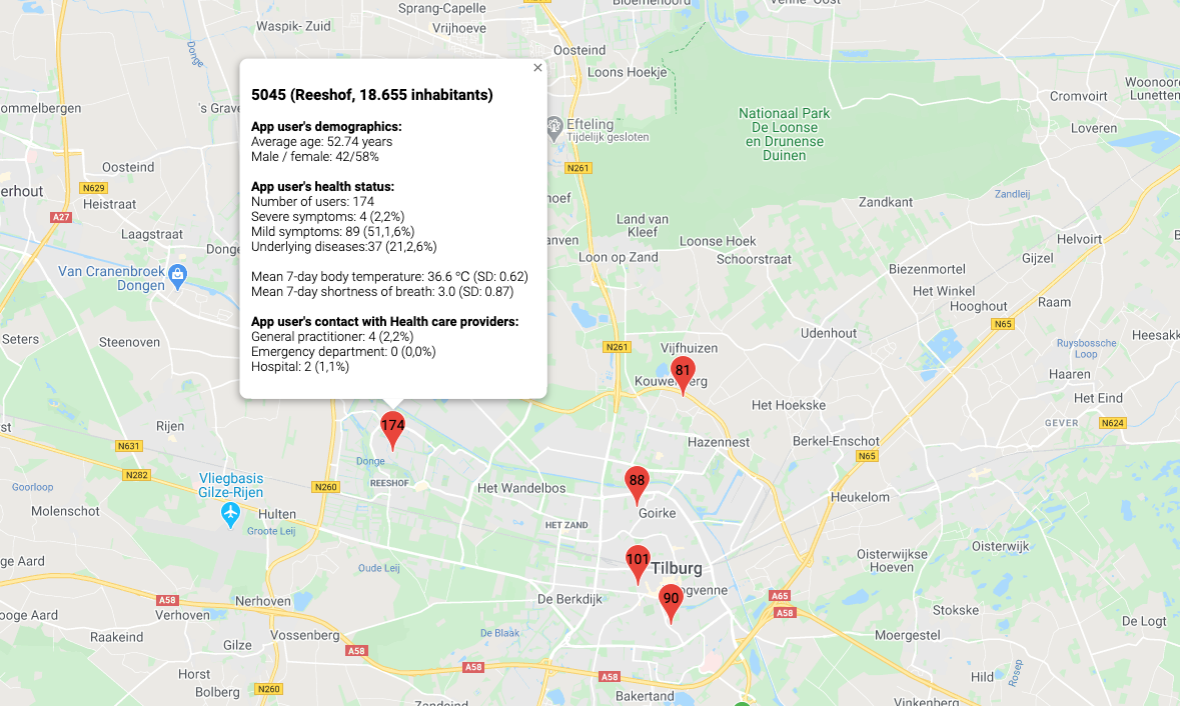
An example of applying the data from our app to the city of Tilburg for the postal codes with the highest number of app users. Per postal code, data on users, health status, and contact with health care providers are available (map created by using the Google Maps application programming interface).

Moreover, the data can be used to create a (near) real time COVID-19 map for the city of Tilburg by applying the data to a Google Maps overview through the Google Maps API (Google; Figure 5).

**Table 4:** An example of combined user data, health status, and contact with health care providers for the postal codes with the highest number of app users.

Postal code	Users, n	Age, mean (SD)	Underlying diseases, n	Mild symptoms, n	Severe symptoms, n	Tested positive^a^, n	General practitioner, n		Emergency department, n		Hospital, n	
							Contact	Follow-up	Contact	Follow-up	Contact	Follow-up
5045	174	52.74 (11.51)	37	89	4	12	4	4	0	0	2	2
5038	101	54.08 (15.52)	14	49	3	8	1	1	0	0	0	0
5021	90	49.00 (15.55)	22	49	4	11	0	0	0	0	0	0
5046	88	48.42 (13.20)	18	49	2	6	2	2	0	0	0	0
5011	81	52.59 (17.86)	17	35	5	21	1	1	0	0	0	0

^a^Clinical data from the Elisabeth Twee Steden microbiology laboratory (date range: April 1 to April 20, 2020).

### Secondary Outcomes

#### Satisfaction With the Information Provided Through the App

In total, 718 users answered the question about their satisfaction with the information in the app. Users in all three groups indicated that they were very satisfied with the information (mean 7.93, SD 1.60 in Tilburg City; mean 7.86, SD 1.51 in the Tilburg Region; and mean 8.08, SD 1.49 in the other area).

#### Added Value of Symptom Monitoring Through the App

In total, 671 users answered the question about the added value of monitoring their symptoms by means of an app. Users in all three groups indicated the added value to be high (mean 8.04, SD 1.83 in Tilburg City; mean 7.95, SD 1.65 in the Tilburg Region; and mean 8.14, SD 1.62 in the other area).

#### Contact With Health Care Providers and Follow-Up Actions Performed

In total, 638 users answered the question about any COVID-19–related contact they had with a health care provider in the 7 days after the start of the symptom diary in the app. Overall, 84% of users (n=526) reported not to have contacted a health care provider. In cases where users did report reaching out, 86 out of 102 contacts (84%) were initiated by users reporting severe (n=2) or mild symptoms (n=84). General practitioners were contacted most frequently (n=92), followed by the hospital (n=8) and the emergency department (n=2). On average, 87.75% of all contact with a health care provider led to a physical appointment with that provider (Table 5).

**Table 5:** Contact with health care provider.

Region	Users, n	General practitioner		Emergency department		Hospital	
		Contacts, n	Visits, n (%)	Contacts, n	Visits, n (%)	Contacts, n	Visits, n (%)
Tilburg City	172	21	20 (95)	1	1 (100)	3	2 (67)
Tilburg Region	174	25	22 (88)	0	0 (0)	2	2 (100)
Other	284	46	38 (83)	1	1 (100)	4	3 (75)
Total	638	92	80 (87)	2	2 (100)	8	7 (88)

## Discussion

### Principal Findings

The results of our study demonstrate the effect and use of an app to provide users with COVID-19 education and functionalities for self-management and symptom management through a 7-day diary. With regards to the primary outcome, over 6000 users downloaded the app in the first 2 weeks after it became available through the app stores. Over 5000 users used the self-assessment tool and over 1300 users started the symptom diary. In addition, over 5200 users were willing to share their characteristics (age and gender) and geographical data (first 4 digits of their postal code). Furthermore, the app received positive evaluations related to the satisfaction with the information and the added value of keeping track of symptoms. Next to this, the app provided valuable insights into the care users had consumed by means of contacting health care providers, as well as the follow-up visits to these providers. Finally, the data that was gathered through the app, could be applied to an interactive map, displaying the health status and characteristics of users, and could be associated with the clinical data from the hospital on a postal code level. Even though the numbers in this study are too small, future data might indicate an association between the number of self-reported severe symptoms and the number of people that are actually tested positive for COVID-19. Therefore, self-reported data could help to gain insights with regard to the spread of the virus in a certain region.

To our knowledge, our study is the first to assess the effectiveness of this combination of education (both national and local hospital-specific guidelines) and self-assessment and symptom tracking functionality. We consider this combination of features, content, and the trusted regional health care provider not only as a major strength of the study but also as a truly differentiating factor of the app itself, especially in these challenging times, where according to the World Health Organization (WHO) “we are not just fighting an epidemic, but an infodemic as well.” The WHO defines infodemics as an excessive amount of information about a problem, which makes it difficult to identify a solution (and allows for misinformation, disinformation, and rumors to make its entrance during a health emergency). The number of downloads from outside the Tilburg Region clearly demonstrates people’s need for apps with these kinds of functionalities. Many of the apps that are used during this pandemic focus on just one aspect, for instance data collection through symptom tracking—sometimes even without providing feedback to users on what the data means or allowing them to track their progress. Another strength of the study was demonstrated by the number of people that were willing to enter their (nonmandatory) personal data into the app, even though it was clearly stated that the app was not connected to the hospital.

A limitation on the use of the app is the decline in the number of users that continued using the symptom diary during the course of the week. Such reductions in compliance have been reported before and often have to do with user’s missing out on personalized features such as reminders, coaching, and interpretation of the data (and the relation to short- and long-term goals) [17]. Within the app we did implement reminders, notifications, and direct feedback on the data users provided about body temperature and shortness of breath. This feedback, however, consisted of generic messages such as “your body temperature is within a safe range” and is something that needs to be tailored to a patient’s specific context. Moreover, measuring body temperature demands some effort and is not as easy to answer than questions like “do you have a runny nose?” or “do you cough?” Using a smart device with sensor data could overcome this barrier [18,19]. The fact that about 700 users reported to be satisfied with the information in the app (mean 8 out of 10 score) and rated the symptom diary with a mean 8 out of 10 score as well demonstrated user’s willingness to participate in this kind of functionality.

Another limitation could be the fact that the app was really a self-assessment tool; the data users provided was not shared with health care professionals. Not linking the app to health care providers was a request from the health care providers themselves, as the app could lower the pressure on the health care system by guiding users in their self-assessments and symptom tracking, and urging them to only contact health care facilities when indicated. A final limitation is the fact that we are not sure about the effect that the education and feedback in the app had on user’s reaching out, or not, to health care providers. In other words, who was reassured by the app and did not contact health care providers, and who was triggered by the app to contact one? In both cases the question remains, was this the right thing to do?

### Clinical Implications

Our study demonstrates that eHealth can be implemented rapidly to successfully support people with important and trustworthy information, self-assessment, and symptom tracking functionality. In this case, the project was initiated by the COVID-19 pandemic, bringing together health care providers and a technology provider to create the app. The results from our study might be applied in other countries that are affected by the COVID-19 pandemic as well. Another application could be the use of the app to support people who are recovering from their hospitalization or home isolation, as many of them have suffered in terms of physical or mental fitness. The app could provide trustworthy education, instructions, and exercises to support users. In addition, in this scenario, health care providers and governments benefit as well, as the data that is being captured provides information about the individual patient’s recovery as well as group-level data on their progress. Finally, in future outbreaks of viruses, apps like these could be a valuable solution to share information and gather data to support the people, health care providers, and policy makers.

A next step in the development of solutions like these, could be more personalized information toward the users. In the COVID-19 case, for instance, users can get specific information that is related to their own health based on their own symptoms or underlying diseases, in combination with the health status (symptoms) in their postal code. From a health care provider’s and policy maker’s perspective this data can be valuable as well, as it could predict the pressure on the (local) health care facilities or the possible sources of infection, based on real time data instead of near real time or even older information from health care provider electronic health records. Even though our study demonstrated that only a small set of anonymous data is enough to start to add value for all stakeholders, linking to more specific data (for instance personal health records or laboratory data) would enable providing an even more personalized advice and monitoring of outcomes over time while taking into account all necessary privacy and security measures. Of course, the real value of the app and the data depends on the number of people that download and use it; the bigger the portion of the population, the better the data represents them.

### Future Research

An important aspect of future research could be the user’s needs, especially when it comes down to their willingness to use the symptom diary. Diaries like these have been successfully applied in apps for patients with chronic diseases, in which patients reported the feeling of safety that their own physician was linked to the data [20]. In addition, the data to be collected should be discussed between health care providers and users; what is the data that health care providers need (and in what frequency) for a reliable advice versus what is the data that users are willing to share from a privacy or workload perspective? The question is, what is the optimal balance? Finally, the requirements for the graphical presentation of the data should be assessed by taking into account the preferences from, for instance, health care providers, policy makers, and public health institutions; whereas, the data itself might be valuable to each of the stakeholders, but the preferences for visualization might differ.

### Conclusion

Our study demonstrated the successful implementation and use of an app with COVID-19 education, self-assessment, and a 7-day symptom diary. Overall, app users were satisfied with the information supplied through the app and appreciated its functionality. Data collected with the app were successfully applied to an interactive map, displaying postal code–specific demographics, health status, and health care consumption. In addition, we were able to link the data to COVID-19 screening results from the hospital’s microbiology laboratory, indicating an association between app users reporting severe symptoms and the number of patients that were tested positive for COVID-19 in the lab. This data could be used to support policy makers and health care providers to get valuable insights in the regional distribution of infection load and health care consumption.

## Appendix

Multimedia Appendix 1 Body temperature 7-day monitoring results and 7-day shortness of breath monitoring results.

## Abbreviations

API: application programming interface
COVID-19: coronavirus disease
eHealth: electronic health
ETZ: Elisabeth Twee Steden
RIVM: Dutch Health Authority
STROBE: Strengthening the Reporting of Observational Studies in Epidemiology
