# Supplementary material for: Using eHealth to Support COVID-19 Education, Self-Assessment, and Symptom Monitoring in the Netherlands: Observational Study
Source: JMIR Mhealth Uhealth. 2020 Jun 23;8(6):e19822. doi: 10.2196/19822 (PMC7313382; doi:10.2196/19822)
Supplement: Multimedia Appendix 1 [file mhealth_v8i6e19822_app1.pdf]

Using eHealth to support COVID-19 education, self-assessment and symptom monitoring. An observational study in The Netherlands.

Multimedia appendix 1

7-day body temperature monitoring results

Body temperature	Day 1	Day 2	Day 3	Day 4	Day 5	Day 6	Day 7
Tilburg city, mean (SD)	36.74 (0.63) n=378	36.67 (0.62) n=204	36.66 (0.60) n=164	36.63 (0.58) n=139	36.61 (0.53) n=120	36.62 (0.53) n=121	36.64 (0.58) n=119
Tilburg region, mean (SD)	36.81 (0.63) n=338	36.74 (0.58) n=202	36.77 (0.64) n=169	36.73 (0.58) n=1135	36.79 (0.62) n=121	36.78 (0.49) n=110	36.76 (0.51) n=116
Other, mean (SD)	36.88 (0.64) n=642	36.89 (0.62) n=317	36.87 (0.59) n=230	36.85 (0.57) n=195	36.88 (0.53) n=166	36.89 (0.50) n=153	36.75 (0.54) n=137
Total (n)<br>% from total	1378<br>100%	736<br>53%	574<br>42%	479<br>35%	416<br>31%	390<br>28%	378<br>27%

7-day shortness of breath monitoring results

Shortness of breath	Day 1	Day 2	Day 3	Day 4	Day 5	Day 6	Day 7
Tilburg city, mean (SD)	3.06 (1.79) n=239	2.88 (1.55) n=143	2.99 (1.79) n=119	3.00 (1.84) n=104	2.55 (1.56) n=85	2.99 (1.90) n=87	2.84 (1.84) n=85
Tilburg region, mean (SD)	2.83 (1.79) n=212	2.63 (1.58) n=131	2.70 (1.56) n=113	2.76 (1.70) n=91	2.99 (1.68) n=72	2.80 (1.69) n=69	2.81 (1.62) n=72
Other, mean (SD)	3.05 (1.75) n=405	3.05 (1.70) n=204	3.13 (1.78) n=147	2.91 (1.67) n=116	2.75 (1.63) n=104	2.80 (1.51) n=98	2.58 (1.51) n=83
Total (n)<br>% from total	856<br>100%	478<br>56%	379<br>44%	311<br>36%	262<br>31%	254<br>30%	240<br>28%
